# Glycolysis, but not Mitochondria, responsible for intracellular ATP distribution in cortical area of podocytes

**DOI:** 10.1038/srep18575

**Published:** 2015-12-18

**Authors:** Shota Ozawa, Shuko Ueda, Hiromi Imamura, Kiyoshi Mori, Katsuhiko Asanuma, Motoko Yanagita, Takahiko Nakagawa

**Affiliations:** 1 TMK project, Medical Innovation Center, Kyoto University, Kyoto, Japan; 2 Pharmacology Research Laboratories II, Mitsubishi Tanabe Pharma Corporation, Saitama, Japan; 3 Department of Internal Medicine, Teikyo University School of Medicine, Tokyo, Japan; 4 Laboratory of Functional Biology, Graduate School of Biostudies, Kyoto University, Kyoto, Japan; 5 Department of Nephrology, Kyoto University Graduate School of Medicine, Kyoto, Japan

## Abstract

Differentiated podocytes, a type of renal glomerular cells, require substantial levels of energy to maintain glomerular physiology. Mitochondria and glycolysis are two major producers of ATP, but the precise roles of each in podocytes remain unknown. This study evaluated the roles of mitochondria and glycolysis in differentiated and differentiating podocytes. Mitochondria in differentiated podocytes are located in the central part of cell body while blocking mitochondria had minor effects on cell shape and migratory ability. In contrast, blocking glycolysis significantly reduced the formation of lamellipodia, a cortical area of these cells, decreased the cell migratory ability and induced the apoptosis. Consistently, the local ATP production in lamellipodia was predominantly regulated by glycolysis. In turn, synaptopodin expression was ameliorated by blocking either mitochondrial respiration or glycolysis. Similar to differentiated podocytes, the differentiating podocytes utilized the glycolysis for regulating apoptosis and lamellipodia formation while synaptopodin expression was likely involved in both mitochondrial OXPHOS and glycolysis. Finally, adult mouse podocytes have most of mitochondria predominantly in the center of the cytosol whereas phosphofructokinase, a rate limiting enzyme for glycolysis, was expressed in foot processes. These data suggest that mitochondria and glycolysis play parallel but distinct roles in differentiated and differentiating podocytes.

Podocyte dysfunction is a common feature of chronic kidney disease. Understanding how these cells maintain their normal function may be the first step in preventing podocyte injury. Normal podocytes have many protrusions from major processes, called foot processes, which interdigitate with foot processes from neighboring cells. A number of proteins on podocyte foot processes form a slit diaphragm and function as a filtration barrier to produce primary urine from blood. This unique podocyte structure is maintained predominantly by the array and distribution of actin filaments. In particular the phosphorylation-mediated signaling mechanism was found to be a mechanism to regulate this cytoskeleton. For example, nephrin is a major protein in the formation of slit membranes in foot processes, with nephrin phosphorylation required to recruit the adaptor protein Nck to assemble actin filaments^1^, suggesting that a substantial level of ATP is required to maintain podocyte structure and function. However, the precise energy metabolism in these cells remains unknown.

The mitochondrion is a high output ATP generator in cells, with many somatic cells relying on mitochondria for their energy supply. By contrast, glycolysis also contributes to energy production under certain circumstances. While this metabolic pathway is a less efficient producer of ATP compared with mitochondria, glycolysis has several advantages. In a couple of cell types, mitochondria exhibit maximum performance constitutively and therefore are unlikely to produce additional energy when needed^2^. By contrast, the glycolytic pathway can be further enhanced to meet cell needs, particularly when mitochondrial function is inhibited^2^. Moreover, in addition to generating energy, glycolysis produces side products, including amino acids, nucleic acid, and lipids^3^. These side products are likely involved in maintaining tumor integrity and/or survival in cancer cells^4^.

Several studies have explored that a couple of cell types have their own unique system for energy metabolism. Nervous system is an example, in which astrocytes are highly dependent on the glycolytic system whereas neurons depend on mitochondria, but not on glycolysis^5^,^6^. In turn, glycolysis is dominant in endothelial cells^7^. Especially, in endothelial tip cells, which take the lead in navigation but do not proliferate at the vascular forefront during vascular sprouting^8^,^9^, ATP was found to be unevenly distributed inside endothelial cells, where glycolysis, but not mitochondrial oxidative phosphorylation (OXPHOS) contributes to local ATP production in cortical areas^7^. Taken together, each cell types possess their own system for energy profile. How OXPHOS and glycolysis are utilized could depend on metabolic needs in individual cell types.

Podocytes have been shown to utilize both OXPHOS in mitochondria and glycolysis to produce ATP^10^. However, the precise role of each system in podocytes remains unknown. This study therefore evaluated the roles of these two distinct systems in undifferentiated and differentiated podocytes, as well as their regulation of intracellular ATP distribution.

## Results

### ATP depletion causes actin derangement and cell death in podocytes

**Figure 1:**
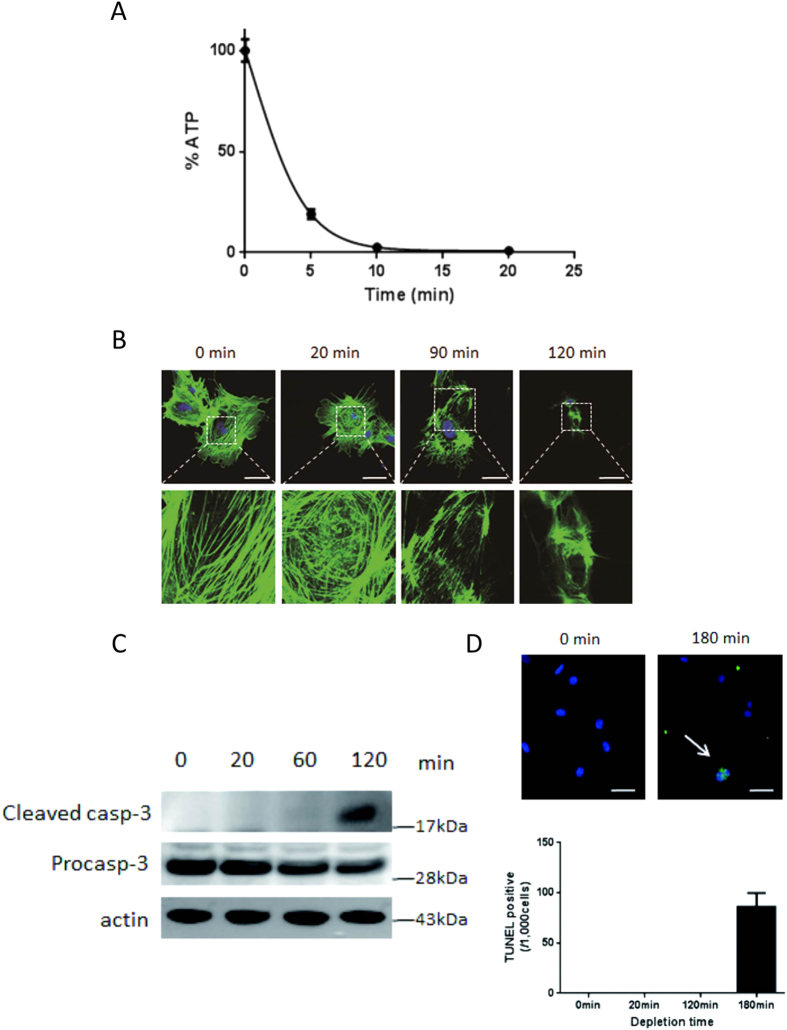
ATP depletion disrupts actin cytoskeleton and induces apoptosis. Incubation of cultured mouse differentiated podocytes with 100 mM 2-deoxyglucose (2-DG) and 10 μM antimycin rapidly depleted intracellular ATP within 15 min (**A**). ATP depletion induced a granular pattern of phalloidin-labeled actin at 20 min compared with actin stress fibers under normal conditions. Subsequently, actin distribution became markedly deranged, with loss of stress fibers at 90 min, and complete disruption at 120 min (**B**). Cleaved caspase 3, an activation marker of caspase 3, was evident at 120 min in the cropped gel (**C**) followed by the appearance of terminal deoxynucleotidyl transferase-mediated dUTP-biotin nick end-labeling (TUNEL) positive cells (arrow) at 180 min (**D**). All blots in the each photo were run under the same experimental conditions. Size markers indicate the cropped level in each figure. Full length gels and blots for each figure are shown in Supplemental Figure 2. Bar, 50 μm.

We first examined the effects of ATP depletion on the actin network in cultured mouse podocytes (Fig. 1A). Treatment of podocytes with 100 mM 2-deoxyglucose (2-DG) and 10 μM antimycin reduced their ATP content to about 20% within 5 min, and to 0% within 15 min. Using phalloidin-labeled actin, we found that ATP depletion appeared to induce a granular actin pattern at 20 min, whereas actin stress fibers were observed under normal conditions. ATP depletion subsequently resulted in marked derangement of actin distribution, with loss of stress fibers at 90 min and completely disruption at 120 min (Fig. 1B). Caspase 3 was activated at 120 min, followed by the appearance of apoptotic cells at 180 min (Fig. 1C,D).

### Mitochondrial OXPHOS and glycolysis occur in podocytes

**Figure 2:**
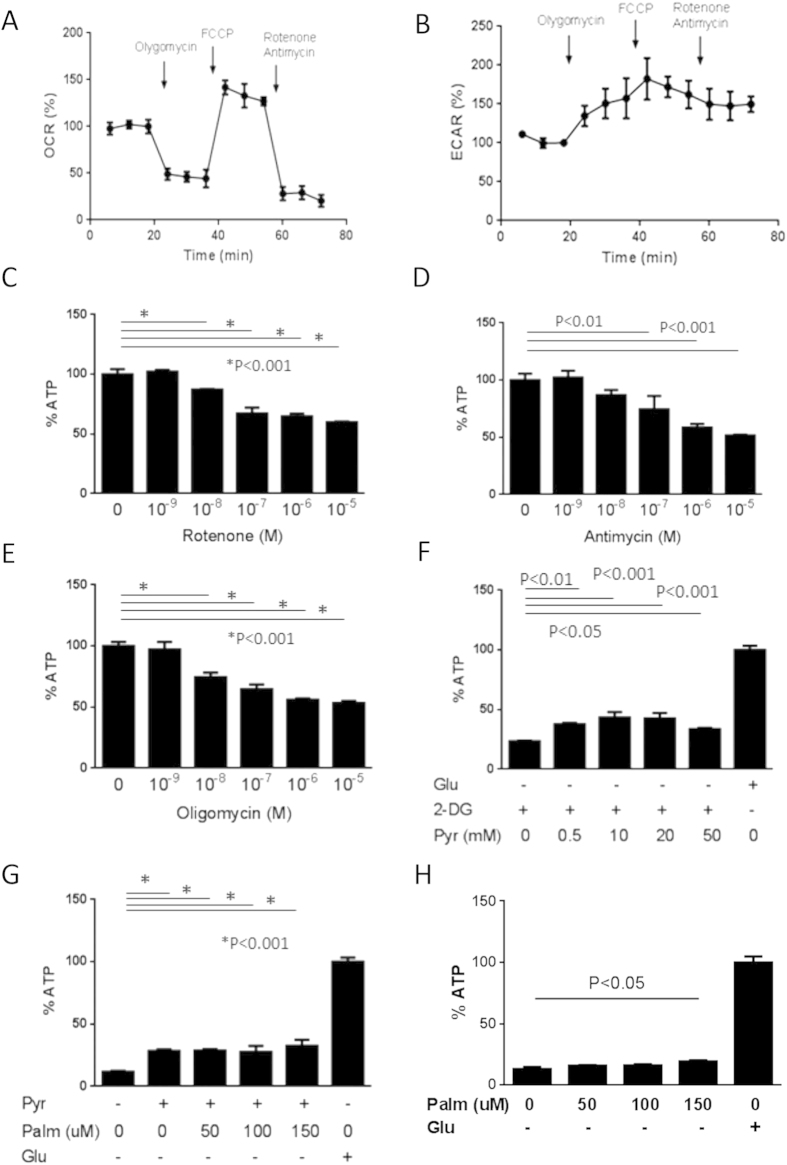
Sources of ATP by mitochondrial respiration and glycolysis. A flux analyzer showed that 1 μM oligomycin reduced oxygen consumption rate (OCR) to 50% compared with baseline conditions (**A**) whereas 2 μM carbonyl cyanide-p-trifluoromethoxyphenylhydrazone (FCCP) increased OCR (**A**) and extracellular acidification rate (ECAR) (**B**) to 142% and 182%, respectively, of control levels, and 2 μM FCCP plus 1 μM rotenone and 1 μM antimycin reduced OCR by 70% (**A**). Dose responses to rotenone (**C**) antimycin and oligomycin showed that all three blockers significantly and dose-dependently reduced intracellular ATP levels by 50% at 1 h (**D,E**). Effects of 2-DG and pyruvate on podocyte concentrations of ATP are shown (**F**). 2-DG in the absence of glucose reduced ATP levels to 25% of control condition at 1 h, whereas adding pyruvate dose-dependently increased ATP level, with 10 mM pyruvate optimally increasing intracellular ATP level. Palmitate had no effect on ATP production (**G**). 50–150 μM palmitate had no effect in the presence of pyruvate but the absence of glucose (**G**) while 150 μM palmitate slightly but significantly increased ATP production in the absence of pyruvate/glucose (**H**).

To investigate bioenergetic profile in normal podocytes, we measured oxygen consumption rate (OCR) and extracellular acidification rate (ECAR) using a Flux analyzer. Incubation of cells with the complex V inhibitor oligomycin reduced OCR to 50% compared with untreated cells, indicating that 50% of cellular oxygen consumption was coupled to ATP synthesis (Fig. 2A). Carbonyl cyanide-p-trifluoromethoxyphenylhydrazone (FCCP), which uncouples electron transport from ATP generation, increased OCR and ECAR to 142% and 182%, respectively, of control levels (Fig. 2A,B), whereas FCCP with rotenone and antimycin reduced OCR by 70% (Fig. 2A).

To assess the contribution of mitochondrial OXPHOS to podocyte energy status, we evaluated the dose dependent responses of podocytes to rotenone, which blocks complex I and therefore interferes with mitochondrial respiration; antimycin, which inhibits mitochondrial complex III; and oligomycin. All three agents significantly and dose-dependently reduced intracellular ATP levels 50% at 1 h (Fig. 2C–E). Given the fact that blocking OXPHOS compensatory increases ATP production by glycolytic activation, these data suggest that mitochondrial OXPHOS contributes to more than 50% of ATP production in podocytes.

To examine the role of glycolysis in podocyte production of ATP, cells were treated with 2-DG plus pyruvate^2^. In the absence of exogenous glucose, 2-DG reduced ATP levels to 25% of control condition at 1 h. Adding pyruvate dose-dependently increased ATP level, with maximal intracellular ATP level observed in the presence 10 mM pyruvate (Fig. 2F). These data suggest that the addition of pyruvate could maintain mitochondrial respiration in the presence of 2-DG but no glucose. That is, glycolysis was likely blocked, but mitochondrial OXPHOS was maintained. We also assessed the effects of palmitate, a free fatty acid and another substrate for mitochondrial respiration. Palmitate had no effect in the presence of pyruvate but the absence of glucose (Fig. 2G) while 150 μM palmitate slightly but significantly increased ATP production in the absence of pyruvate/glucose (Fig. 2H). These data suggests that fatty acids are not major substrates for ATP production in podocytes in the presence of pyruvate, but can be utilized in the absence of other energy sources.

### Effect of blocking either OXPHOS or glycolysis on cell shape and migration at identical ATP levels

**Figure 3:**
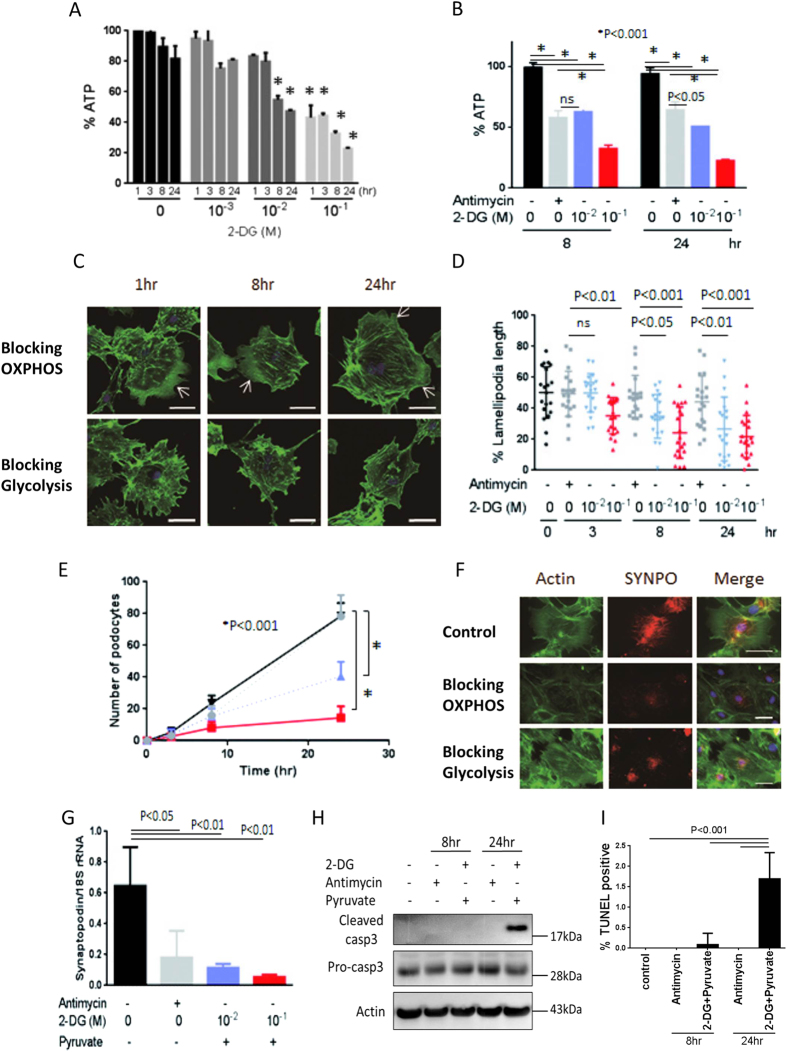
Effect of blocking OXPHOS or glycolysis on cell shape migration and apoptasis. Dose- and time-dependent effects of 2-DG on ATP level. 10 mM 2-DG mildly and significantly reduced ATP level at 8 and 24 h (**A**). Intracellular ATP level following incubation with 10 mM 2-DG for 8 h was similar to that with 10 M antimycin (**B**). Assays using phalloidin-labeled actin filaments, showing that 10 mM 2-DG plus pyruvate prevented lamellipodia formation (arrow), whereas 10 μM antimycin had little effect on podocyte cell shape (**C**). Lamellipodia length was significantly reduced following incubation with 10 mM or 100 mM 2-DG plus 10 mM pyruvate after 8 and 24 h, but not with 10 μM antimycin (**D**). In contrast, 10 μM antimycin did not significantly alter lamellipodia formation (**D**). Both 10 mM (purple dotted line) and 100 mM (red dotted line) 2-DG significantly reduced podocyte migration at 24 h, whereas 10 μM antimycin (gray dotted line) had no effect (**E**). 2-DG plus pyruvate, as well as antimycin reduced synaptopodin (SYNPO) immunofluorescence (red); the green signal indicates actin (**F**). Synaptopodin mRNA expression measured by qPCR was significantly reduced by 2-DG plus pyruvate and by antimycin (**G**). 100 mM 2-DG induces cleaved caspase-3 (in the cropped blots) (**H**). and increases in the number of TNNEL-positive cells (**I**) in the cultured podocytes. However, antimycin did not show any response on these parameters (**H,I**). Bar, 50 μm. All blots in the each photo were run under the same experimental conditions. Size markers indicate the cropped level in each figure. Full length gels and blots for each figure are shown in Supplemental Figure 2.

First, we sought the conditions under which either OXPHOS or glycolysis was blocked to generate identical ATP levels. The effects of 2-DG dose and incubation time on intracellular ATP levels were examined. 2-DG was found to significantly reduce ATP level at 8 and 24 h (Fig. 3A), with the level of intracellular ATP observed after treatment with 2-DG for 8 h identical to that observed in cells treated with antimycin (Fig. 3B). We then compared the effects of 2-DG and antimycin on podocyte migration and lamellipodia length. Using actin filaments labeled with phalloidin, we found that 2-DG plus pyruvate prevented the formation of lamellipodia, whereas antimycin had no effect on podocyte shape (Fig. 3C). Lamellipodia formation was assessed quantitatively by calculating percent lamellipodia length and area relative to control cells. Blocking glycolysis significantly reduced percent lamellipodia length (Fig. 3D) and area (data not shown) at 8 and 24 h, whereas blocking mitochondria did not result in significant changes in lamellipodia formation (Fig. 3D). Evaluations of podocyte migration showed that blocking glycolysis with both 10 mM and 100 mM 2-DG significantly reduced podocyte migratory ability, whereas blocking mitochondria had no effect (Fig. 3E). These findings indicate that blocking glycolysis, even partially, can inhibit lamellipodia formation and cell migration.

We also assessed the effects of blocking either glycolysis of mitochondria on the expression of synaptopodin, a key molecule in differentiated podocytes. Interestingly, synaptopodin expression was significantly reduced, both by blocking glycolysis and by incubation with antimycin (Fig. 3F,G), suggesting that this differentiation marker is regulated by both systems.

In terms of apoptosis, blocking glycolysis appeared to induce level of cleaved caspase-3 (Fig. 3H) and increase in TNNEL-positive cells (Fig. 3I) in the cultured podocytes. However, antimycin failed to induce apoptosis.

As 100 mM 2DG produces hyperosmolarity, the phenotypic changes of podocytes could be due to osmotic pressure. We therefore examined the effect of 100 mM Mannitol. However, we found that 100 mM Mannitol had no effect on cell shape, migration and apoptosis in the podocytes (Supplemental Figure 1).

**Figure 4:**
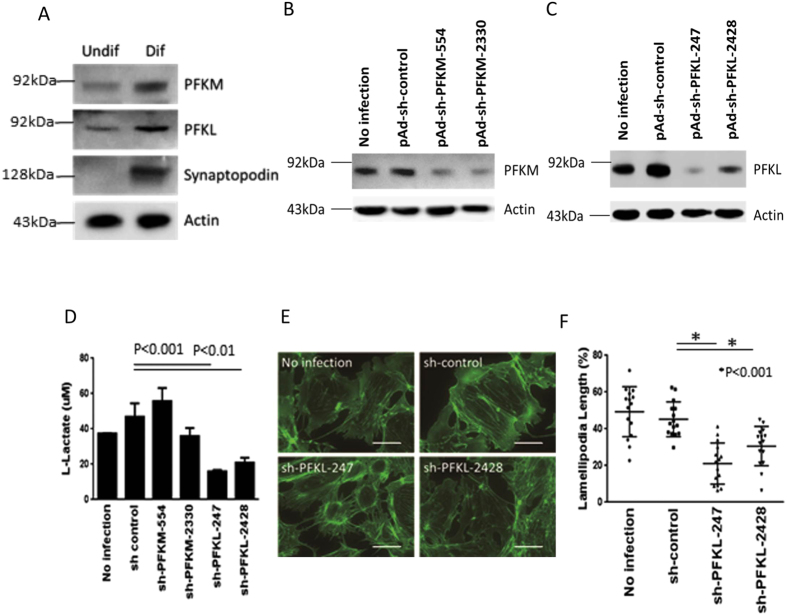
Effect of phosphofructokinase knockdown on cell shape. Two types of phosphofructokinases (PFKs, PFK-M and PFK-L) are expressed in undifferentiated and differentiated podocytes (**A**). Transduction with adenovirus carrying shRNA for either PFK-M or PFK-L reduced their respective expression (**B,C**). Reduced PFK-L, but not PFK-M, expression significantly reduced intracellular L-lactate concentration (**D**). Phalloidin-labeled actin, showing that blocking glycolysis with PFK-L knockdown impaired lamellipodia formation (**E**), and quantification (**F**). Bar, 50 μm. Cropped gels/blots are shown in (**A–C**). Each blot was run under the same experimental conditions. Size markers indicate the cropped level in each figure. Full length gels and blots for each figure are shown in Supplemental Figure 2.

To confirm the effect of blocking glycolysis, we knocked down a key genes encoding phosphofructokinase (PFK), a rate limiting enzyme, in the glycolytic pathway. We found that these cultured podocytes expressed two types of PFKs, PFK-M and PFK-L, and that the expression of both was upregulated during the differentiation process (Fig. 4A). Adenoviruses carrying shRNAs for PFK-M (PFKM-554, PFKM-2330) or PFK-L (PFKL-247, PFKL-2428) were capable of knocking down the expression of these two genes (Fig. 4B,C). Interestingly, a reduction in PFK-L, but not PFK-M, could lower intracellular L-lactate concentrations (Fig. 4D), suggesting that PFK-L plays a key role in the glycolytic pathway in the differentiated podocytes. Similar to the effect with pharmaceutical intervention, blocking glycolysis with PFK-L knockdown resulted in impairment of lamellipodia formation (Fig. 4E,F).

### Glycolysis contributes to local ATP production in lamellipodia

**Figure 5:**
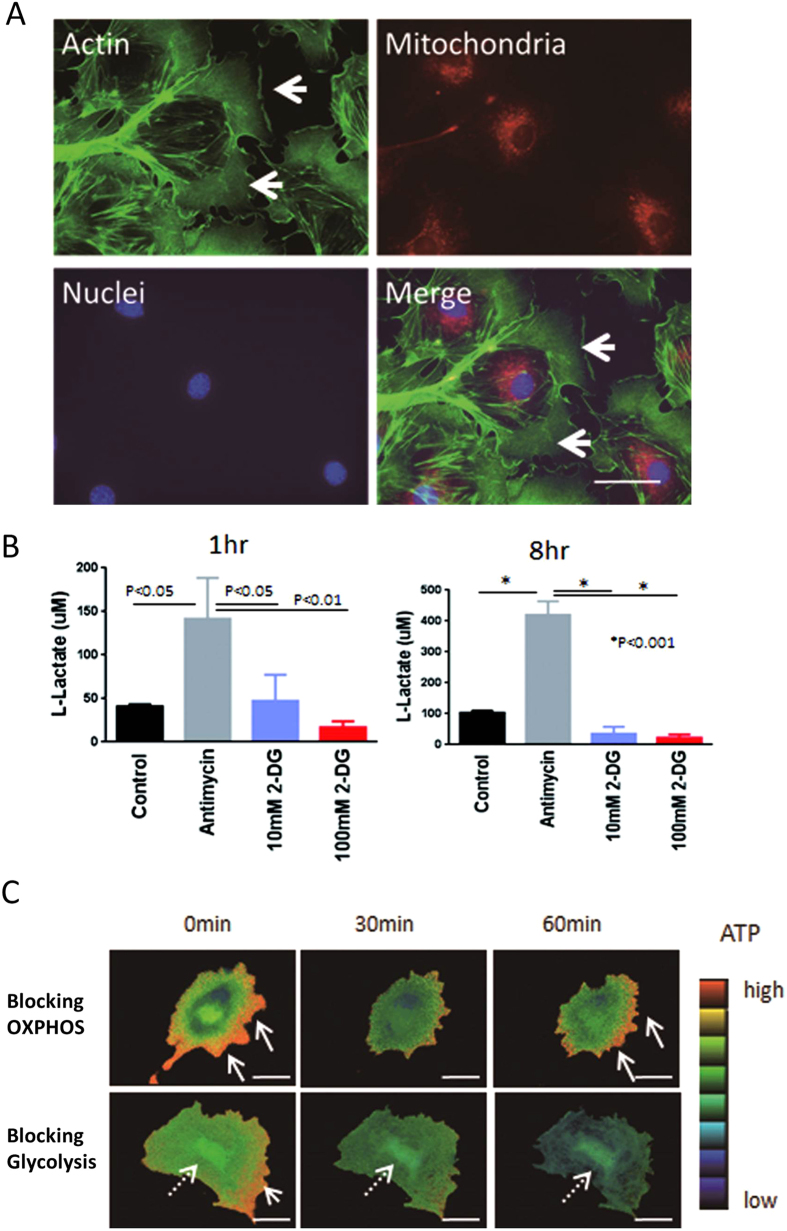
Glycolysis contributes to the local ATP production for lamellipodia. Mitochondria (red color) are predominantly located in the center of the cell body, but spatially dissociated from lamellipodia (arrow) (**A**). Blocking OXPHOS with antimycin increased intracellular lactose levels at 1 and 8 h (**B**). Monitoring of intracellular ATP distribution using genetically encoded fluorescence resonance energy transfer (FRET)-based ATP indicators. Antimycin reduced ATP levels in the center of the cytosol, while ATP level was maintained at cortical sites, where the lamellipodia were located (arrow). In contrast, blocking glycolysis reduced ATP level in the cortical area but not around the nucleus (dotted arrow) (**C**). Bar, 50 μm.

We wondered why mitochondria did not contribute to lamellipodia formation. Then, we examined the spatial associations of mitochondria and lamellipodia in differentiated podocytes. Most mitochondria were present in the center of the cell body and were spatially dissociated from the lamellipodia (Fig. 5A), indicating that the lamellipodia may not be regulated by mitochondria and may therefore depend on glycolysis. To assess the role of glycolysis in lamellipodia, the functional association between glycolysis and mitochondria was examined. Blocking OXPHOS with antimycin increased extracellular lactate levels at both 1 and 8 h (Fig. 5B), suggesting that glycolytic activity is maintained and even enhanced by blocking mitochondrial OXPHOS. We then assessed whether ATP production by glycolysis could contribute to the lamellipodia formation in these cells. To monitor intracellular ATP distribution, we utilized genetically encoded fluorescence resonance energy transfer (FRET)-based ATP indicators, employing the ε subunit of bacterial FoF1-ATP synthase^11^. Blocking mitochondria with antimycin reduced ATP levels in the center of the cytosol, while ATP levels were maintained in the cortical area of the cells, in which the lamellipodia were located (Fig. 5C). In contrast, blocking glycolysis lowered ATP levels in the cortical area but had little effect on ATP levels around the nucleus (Fig. 5C). Taken together, these findings indicate that glycolysis, which was preserved when mitochondria were blocked, is responsible for the ATP production necessary for lamellipodia formation in podocytes.

### Nitric oxide blocks OXPHOS but does not alter cell shape or cell migratory ability

**Figure 6:**
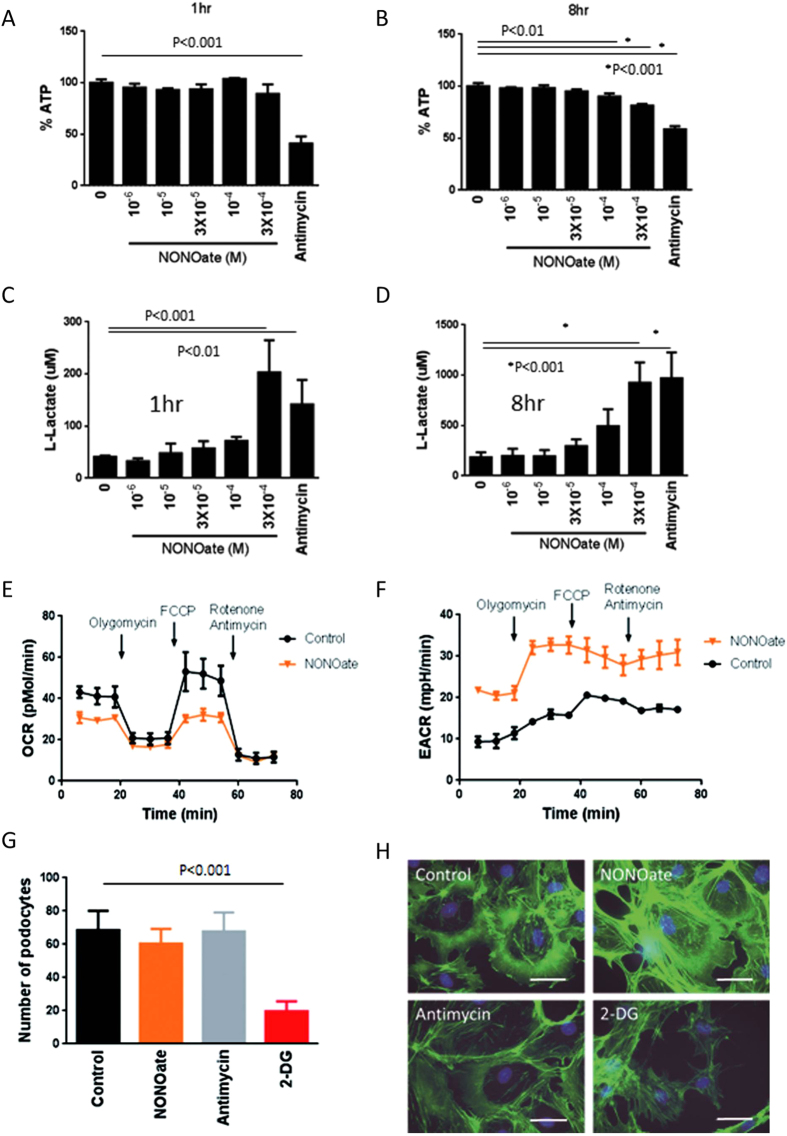
Nitric oxide blocks OXPHOS but does not alter cell shape or cell migratory ability. (Z)-​1-​[N-​(2-​aminoethyl)-​N-​(2-​ammonioethyl)amino]diazen-​1-​ium-​1,​2-​diolate (NONOate) (3 × 10^−4^ M) significantly reduced intracellular ATP level in differentiated podocytes at 8 h (**B**) but not at 1 h (**A**). NONOate dose-dependently increased L-lactate concentration at 1 and 8 h (**C,D**). Flux analyzer showing that NONOate reduced OCR (**E**) while increasing ECAR (**F**) compared with control. NONOate did not alter cell migration (**G**). Similar to antimycin, NONOate had no effect on lamellipodia formation, which was inhibited by 2-DG (H). Bar, 50 μm.

Nitric oxide (NO) has been reported to inhibit OXPHOS and activate the glycolytic pathway in other cell types, including astrocytes^12^. We therefore evaluated the effects of NO in podocytes. While it had minor effects at 1 h (Fig. 6A), 3 × 10−4 M (Z)-​1-​[N-​(2-​aminoethyl)-​N-​(2-​ammonioethyl)amino]diazen-​1-​ium-​1,​2-​diolate (NONOate) significantly reduced intracellular ATP level at 8 h in differentiated podocytes (Fig. 6B). Simultaneously, NONOate dose-dependently activated the glycolytic pathway in these cells, increasing L-lactate concentration at 1 and 8 h (Fig. 6C,D). ECAR was remarkably higher with NONOate compared with control (Fig. 6F), while reducing OCR (Fig. 6E). These data indicated that the effects of NO on OXPHOS and the glycolytic pathway in podocytes were similar to the effects of antimycin. Then we examined the effect of NONOate on podocyte shape and migratory ability. As expected, NONOate suppressed OXPHOS, but did not alter migration ability or lamellipodia formation (Fig. 6G,H).

### Roles of OXPHOS and glycolysis in podocyte differentiation

**Figure 7:**
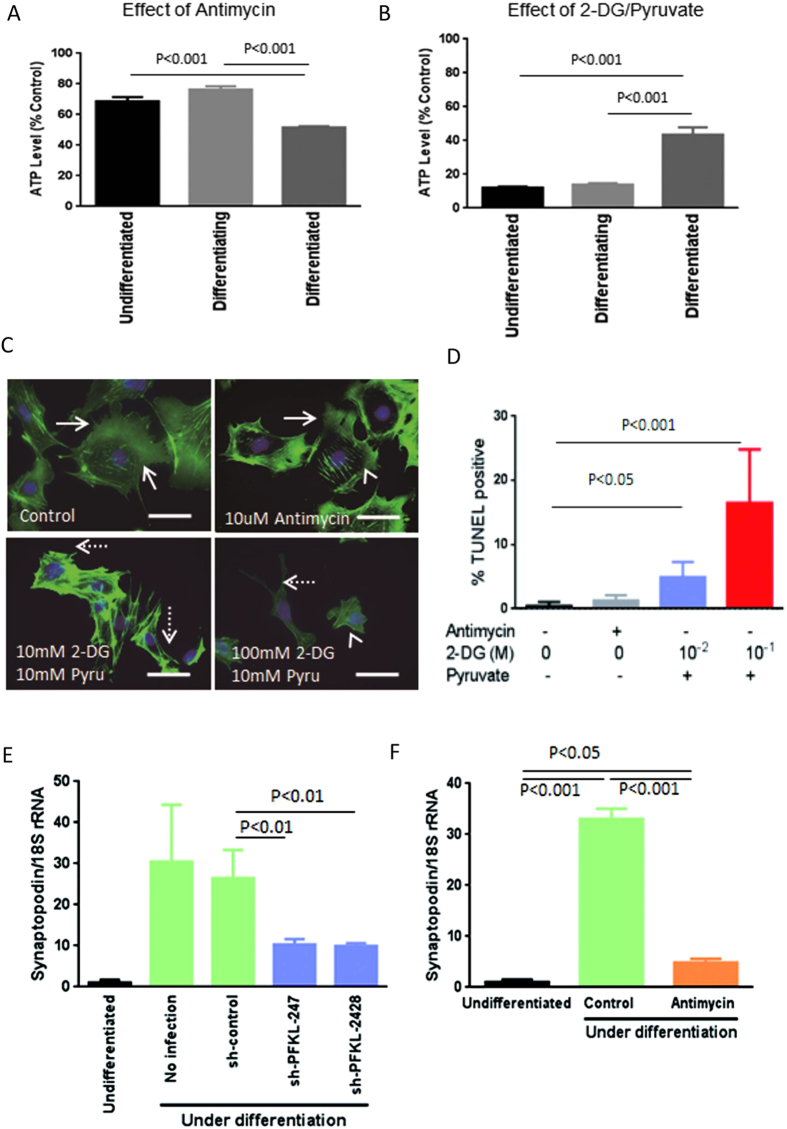
Roles of OXPHOS and glycolysis in differentiating podocytes. Antimycin reduced intracellular ATP levels 31% in undifferentiated cells and 24% in differentiating podocytes, but 49% in differentiated podocytes (**A**). In contrast, blocking glycolysis with 2-DG and pyruvate reduced ATP levels by 88% in undifferentiated cells, 86% in differentiating podocytes and 56% in differentiated podocytes (**B**). 2-DG with pyruvate inhibited lamellipodia formation, whereas antimycin had a minor effect (**C**). White arrows indicate lamellipodia formation whereas dotted arrows show a lack of lamellipodia formation in C. Blocking glycolysis significantly increased the number of TUNEL positive differentiating podocytes, whereas antimycin had no effect (**D**). sh-PFKL247 and sh-PFKL-2428 significantly reduced synaptopodin expression in differentiating podocytes in nonpermissive conditions (**E**). Antimycin also reduced synaptopodin mRNA expression in differentiating podocytes (**F**). Bar, 50 μm.

We next examined the involvement of OXPHOS and glycolysis in podocyte differentiation. Undifferentiated podocytes maintained at growth permissive conditions at 33 °C were transferred to to nonpermissive conditions at 37 °C for 3 days to prepare the differentiating cells and for 7 days to render the differentiated podocytes. After these cells were exposed to antimycin or 2-DG plus pyruvate for 1 h, ATP levels were measured. Interestingly, antimycin reduced intracellular ATP levels by only 31% in undifferentiated cells and by only 24% in differentiating cells, compared with a reduction of 49% in differentiated podocytes (Fig. 7A). In contrast, blocking glycolysis with 2-DG and pyruvate reduced ATP levels by 89% in undifferentiated cells and 86% in differentiating podocytes, compared with 56% in differentiated podocytes (Fig. 7B). Assessments of cell shape showed that blocking glycolysis eliminated lamellipodia formation, whereas antimycin had a minor effect (Fig. 7C). The effects of these agents on cell apoptosis were also examined using terminal deoxynucleotidyl transferase-mediated dUTP-biotin nick end-labeling (TUNEL) assays. Blocking glycolysis significantly induced apoptosis at 72 h in differentiating podocytes, while antimycin had minor effects. Synaptopodin is a key protein induced during differentiation but not expressed in undifferentiated podocytes^13^,^14^. We therefore examined the effects of ATP generators on synaptopodin expression in differentiating podocytes. Although synaptopodin was not detected in undifferentiated podocytes (Fig. 4A), it was induced in differentiating podocytes on 7 days under nonpermissive conditions (Fig. 7E,F). Interestingly, both glycolysis and mitochondria were found to be involved in synaptopodin expression, as blocking either significantly reduced its expression (Fig. 7E,F). These findings suggest that glycolysis may be indispensable for lamellipodia formation and cell survival, whereas synaptopodin expression could be regulated by both systems.

### Role of glycolysis and mitochondria in other cell types

**Figure 8:**
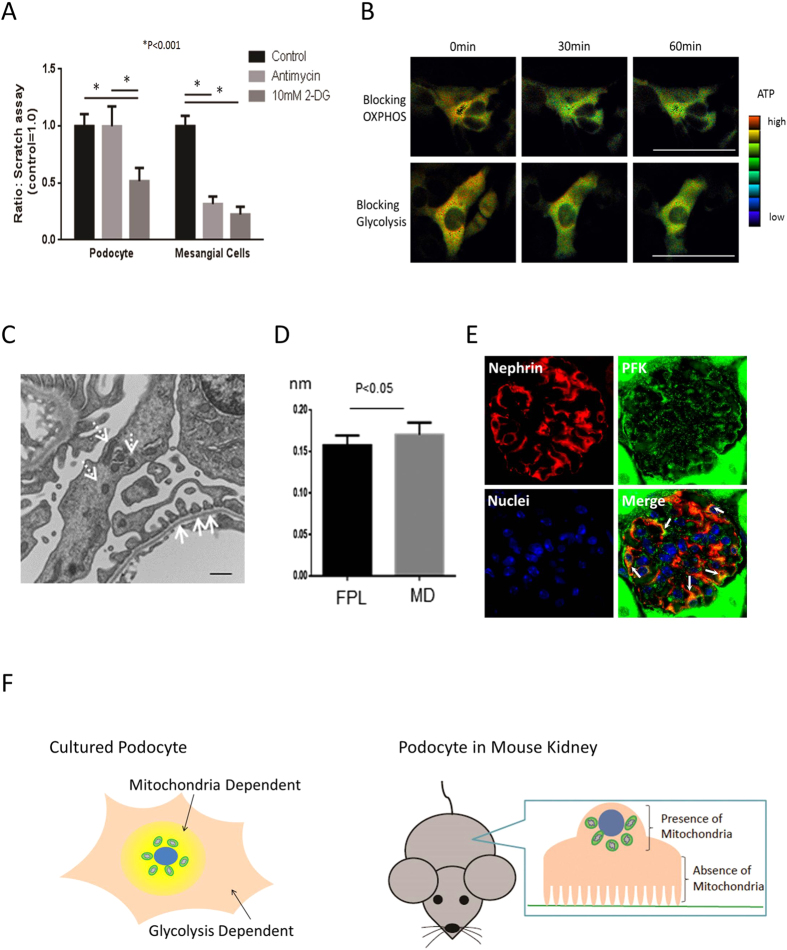
Effects of mitochondria and glycolysis on migration of other type of cells, and intracellular mitochondrial localization in podocytes of adult mice. In contrast to differentiated podocytes, 10 μM antimycin significantly reduced the migration of rat mesangial cells. In contrast, blocking glycolysis impaired cell migration in all cell types (**A**). Intracellular ATP level are likely reduced in the whole area of mesangial cell and its distribution are not altered by either antimycin or 10 mM 2DG/pyruvate (**B**). Bar, 50 μm. Electron micrograph showing that mitochondria (arrow) are predominantly located in the cell body and major processes, but not in foot processes, of adult mouse podocytes (**C**). Bar, 500 nm. Mitochondrial diameter (MD) is significantly greater than the length of foot processes (FPL) (**D**). Immunofluorescence shows that PFK is overlapped with nephrin (indicated by white arrow) in the mouse glomerulus (**E**). Schematic cartoons showing ATP distribution from mitochondria and glycolysis in cultured podocytes and the location of mitochondria in podocytes of mouse kidney are shown (**F**).

To determine whether the effects of blocking glycolysis or mitochondrial respiration on cellular responses were specific to podocytes, we tested the effects of these agents on the migratory ability of mesangial cells, which are another type of kidney cells. In contrast to podocytes, migration of mesangial cells was significantly blocked by either antimycin or 2-DG (Fig. 8A). In terms of intracellular ATP distribution, the effect of antimycin was likely similar to that of 2-DG. ATP level seemed to be reduced in the whole area of cells and as a result, intracellular ATP distribution was not altered by either measures (Fig. 8B).

### Intracellular mitochondrial localization in the podocytes of adult mice

Mitochondria are located in the central area of the cytosol in cultured podocytes (Fig. 6A). We then examined localizations of mitochondria and phosphofructokinase (PFK), a glycolytic enzyme localization, in the podocytes of adult mice. Electron microscopy showed that mitochondria in these cells were predominantly located in the cell body and major processes, but not in the food processes (Fig. 8C). Mitochondrial diameter was significantly greater than foot process width (Fig. 8D). In contrast, PFK was overlapped with nephrin, suggesting that PFK is located in the foot process (Fig. 8E). Finally, schematic cartoons are shown in Fig. 8F.

## Discussion

Mitochondria are high output ATP generators, with many important functions involved in the maintenance of cellular physiology while ATP is also produced by glycolysis. The balance between these two pathways is likely controlled by a sophisticated mechanism, allowing cell survival under various conditions. In cells with a unique structure, mitochondria can move intracellularly to locations required to meet energy demands. Thus, mitochondria are not evenly distributed throughout the entire cell, but must be positioned properly to serve the need of the cells.

Mitochondria in proximal tubular epithelial cells are located near the basement membrane, where ATPases are located. Likewise, neurons, which consist of three distinct structural domains, the cell body (soma), axon and thick dendrites, contain a number of mitochondria in pre-and post-synaptic terminals, perhaps because metabolic demand is particularly high in synaptic terminals^15^. Neurons were shown to be equipped with a sophisticated delivery system for mitochondria, meeting the unique metabolic demands of these cells. This notion could account for why axonal regions, which have higher levels of activity, have higher mitochondrial density^16^,^17^.

Similar to neurons, podocytes exhibit a unique cytoarchitecture, with branching array of foot processes that are essential for glomerular filtration. We initially assumed that mitochondria might play a key role for energy supply in the foot process. However, in contrast to our assumption, we found that mitochondria were not located in the cortical area cultured differentiated podocytes, but in the cytosol around the nucleus. Likewise, mitochondria were not present in foot processes in mouse podocytes, perhaps because the mitochondria were physically larger in size than foot processes. Based on the concept that mitochondria must be positioned properly to serve the needs of the cells, energy metabolism in foot processes may not be supported by mitochondrial respiration, but rather by glycolysis.

Podocyte energy metabolism is covered by two systems, but roles of each remains undetermined yet. Here, we found that glycolysis contributed to apoptosis regulation, cell motility, lamellipodia formation, and local ATP production in the cortical area of cells. In contrast, ATP produced by mitochondria was unlikely involved in lamellipodia formation or cell migration. These results indicate that the intracellular energy profile in the cortical area of these cells and cell survival were regulated by glycolysis whereas energy in the central area was controlled by both glycolysis and mitochondria. Other types of cells likely utilize a similar system. For example, prostate cancer and endothelial cells rely on glycolysis to regulate lamellipodia formation and cell motility, while glycolysis also contributes to endothelial cell proliferation^2^,^7^. On the other hand, mitochondria likely play other critical roles in addition to synaptopodin expression in these cells^18^,^19^.

Mitochondrial respiration was found to account for approximately only 20% of intracellular ATP content in undifferentiated and differentiating cells, but increased to 50% after differentiation was completed. These findings suggest that glycolysis may be a main source for ATP production in the early phase of differentiation process while mitochondrial contribution would be more dominant after differentiated. In turn, other types of cells exhibit different balance of mitochondria with glycolysis. For example, hematopoietic stem cells reside in a hypoxic niche in the bone marrow and rely heavily on anaerobic glycolysis^20^,^21^. However, mitochondrial respiration is likely required for differentiation, as blocking mitochondria by inactivating *Ptpmt*, a gene encoding phosphatase and tensin homolog (PTEN)-like mitochondrial phosphatase, completely prevented cell differentiation^22^. By contrast, human embryonic stem cells rely on mitochondrial OXPHOS in their undifferentiated stage, but require glycolysis during the process of cell differentiation^23^. Therefore, different cells likely utilize different balances of ATP resulting from glycolysis and mitochondrial respiration during and after differentiation. Regarding to this issue, our findings suggest that mitochondria were unlikely involved in the motility of differentiated podocytes, in contrast to mesangial cells, which utilized mitochondrial respiration for cell migration.

Synaptopodin is a proline-rich, actin-associated protein expressed in highly dynamic cell compartments, such as the dendritic spine apparatus of neurons and podocyte foot processes^13^,^14^. This protein is commonly considered as a marker for differentiated podocytes, as it is not expressed in undifferentiated podocytes but emerges during the process of differentiation^13^,^14^. Our results showed that synaptopodin expression was reduced by blocking either mitochondrial respiration or glycolysis in both differentiating and differentiated podocytes, suggesting that both systems regulate synaptopodin expression in these cells, regardless of their stage of development. In differentiated cells, synaptopodin is usually associated with actin filaments^14^, which are present in the cytosol as well as the lamellipodia. These findings suggest that mitochondria may contribute to synaptopodin expression in cytosol, whereas glycolysis may be responsible for synaptopodin expression in lamellipodia. Actin polymerization with synaptopodin is involved in extending protrusions during differentiation^24^, which requires ATP^25^. Both mitochondria and glycolysis could contribute to this differentiation process.

ATP is the ubiquitous energy currency for all living cells, making it very important to precisely understand how ATP contributes to cellular metabolism and how intracellular ATP level is regulated at the single cell level. However, understanding intracellular energy metabolism remains elusive, perhaps because conventional methods of ATP quantification sample entire cell extracts and can only measure average ATP levels in cells, making ATP distribution patterns unclear. This study used genetically encoded fluorescence resonance energy transfer (FRET)-based ATP indicators, employing the ε subunit of bacterial FoF1-ATP synthase^11^. This approach enabled the determination of intracellular ATP delivery by each system.

We clearly demonstrated that ATP was unevenly distributed in podocytes and that local ATP production in cellular compartment was regulated by different systems. Glycolysis was found to be responsible for providing ATP in the cortical area, while mitochondria respiration was responsible for the cytosol around the nucleus. Similarly, endothelial cells utilize glycolysis to provide ATP in the lamellipodia. In fact, cultured endothelial cells showed sparse mitochondria in lamellipodia, where glycolysis was responsible for ATP supply^7^. Each type of cell may have unique systems to cover cellular function, enabling mitochondria to contribute to lamellipodia and cellular motility. Since we found that mitochondria were in part responsible for the migration of mesangial cells, ATP derived from mitochondria may be distributed in the cortical parts of these cells.

We have reported that NO derived from eNOS can act on podocytes, modulating their structure and mitochondrial function in mouse kidneys^26^,^27^. Recent studies examining the mechanism by which NO modulates mitochondrial function found that NO inhibits cytochrome c oxidase and increases glycolytic activation. However, NO response was dependent on cell type. For example, NO rapidly activates the glycolytic pathway in astrocytes, but not in neurons^12^. We therefore examined the role of NO in podocytes. Similar to findings in astrocytes, we found that NO inhibited mitochondrial respiration and activated the glycolytic pathway in podocytes. These data indicate that podocytes respond to NO similar to astrocytes, and NO may be important for podocyte cell integrity and mitochondrial function.

In conclusion, ATP was generated by both mitochondria and glycolysis in differentiating and differentiated podocytes. Each system has its own roles in delivering ATP to target compartments in these cells.

## Materials and Methods

All procedures were performed in accordance with the guidelines of the research center of Kyoto university graduate school of medicine. All experimental protocols were approved by the gene recombination experiments safety committee in the university.

### Chemicals and virus vectors

Oligomycin, rotenone, antimycin and 2-DG were purchased from Sigma Aldrich (St. Louis MO, USA). FCCP was from Abcam (Cambridge, UK) and sodium pyruvate from Nacalai Tesque (Kyoto, Japan). Sodium palmitate (Sigma) was dissolved in 50% ethanol and mixed vigorously with 12.5% fatty-acid-free bovine serum albumin in phosphate-buffered saline (PBS). The sequences of the shRNA expression vectors were designed using RNAi Designer (Life Technologies). These sequences included PFKM-554 forward, 5′-CACCGCTGAATGATCTCCAGAAAGACGAATCTTTCTGGAGATCATTCAGC-3′, and reverse, 5′-AAAAGCTGAATGATCTCCAGAAAGATTCGTCTTTCTGGAGATCATTCAGC-3′; PFKM-2330 forward, 5′-CACCGGACCAGACAGACTTTGAACACGAATGTTCAAAGTCTGTCTGGTCC-3′, and reverse, 5′-AAAAGGACCAGACAGACTTTGAACATTCGTGTTCAAAGTCTGTCTGGTCC-3′; PFKL-247 forward, 5′-CACCGGGCCAAAGTCTTCCTCATCTCGAAAGATGAGGAAGACTTTGGCCC-3′, and reverse, 5′-AAAAGGGCCAAAGTCTTCCTCATCTTTCGAGATGAGGAAGACTTTGGCCC-3′; PFKL-2428 forward, 5′-CACCGCACCTTGAGCATAGACAAGGCGAACCTTGTCTATGCTCAAGGTGC-3′, and reverse, 5′-AAAAGCACCTTGAGCATAGACAAGGTTCGCCTTGTCTATGCTCAAGGTGC-3′; and Control forward, 5′-CACCGCTACACAAATCAGCGATTTCGAAAAATCGCTGATTTGTGTAG-3′, and reverse, 5′-AAAACTACACAAATCAGCGATTTTTCGAAATCGCTGATTTGTGTAGC-3′. Each double-stranded oligonucleotide was cloned into donor vector (pENTR/U6) and transferred to an adenovirus expression vector (pAd/BLOCK-iT-DEST). The recombinant adenovirus backbones were linearized by PacI digestion and transfected into 293A cells for packaging. Viral titers were estimated using an horseradish peroxidase (HRP) conjugated polyclonal antibody to adenovirus hexon (Thermo Fisher Scientific, Waltham, MA). Cellular phenotypes were examined 72 h after infection with shRNA.

### Cells

Conditionally immortalized mouse podocyte cell lines cultured at 33 °C in RPMI-1640 medium with 10% fetal bovine serum (FBS) and 10 U/ml recombinant mouse γ-interferon (IFN) were transferred to non-permissive conditions at 37 °C without γ-IFN for at least 7days^28^. While it is concerned that temperature mediated differentiation process may affect biological response, this tool is currently most widely utilized to study podocytes biology^28^. Rat mesangial cells (CRL-2573) were purchased from ATCC (VA, USA) and cultured in Dulbecco’s modified Eagle’s medium high glucose medium supplemented with 10% FBS.

### Mitochondrial function and glycolysis

Total ATP concentrations in cell lysates were measured with ATP Assay Kit (BioAssay Systems, Hayward, CA, USA) according to the manufacturer’s instructions. Briefly, ATP released from cells immediately reacted with the substrate D-luciferin to produce light in the presence of luciferase. Light intensity was measured using Enspire (Perkin Elmer, Waltham, MA, USA). Differentiated mouse podocyte cells seeded in collagen coated XF96-well microplates at 2 × 10^4^ cells per well were incubated at 37 °C with 5% CO_2_ for several days. One hour after medium was replaced by RPMI 1640 without bicarbonate, the OCR and ECAR were measured with XF96 (Seahorse Bioscience, North Billerica, MA, USA) before and after injection of 1 μM oligomycin, 2 μM FCCP, 1 μM rotenone and 1 μM antimycin. Extracellular L-lactate was measured using an L-lactate assay kit (Cayman Chemical, Ann Arbor, MI, USA) according to the manufacturer’s instructions. Briefly, deproteinated supernatant was oxidated to pyruvate by lactate dehydrogenase, along with the concomitant reduction of NAD^+^ to NADH. NADH reacted with the fluorescent substrate to yield a highly fluorescent product, which was analyzed using Enspire at an excitation wavelength of 535 nm and an emission wavelength of 590 nm.

### Intracellular ATP distribution

As previously reported^11^, mouse podocyte cells infected with retrovirus to produce ATeam1.03 (pQCXIN-ATeam1.03) were incubated with 800 μg/ml G418 to obtain cells stably expressing ATeam1.03. For rat mesangial calls, adenovirus ATeam1.03 was used. The cells were illuminated with a 75-W xenon lamp through 12.5% and 25% neutral density filters. Fluorescence emission from ATeam was imaged using a cooled charge-coupled device (CCD) camera; the exposure times were 500 ms for both cyan fluorescent protein (CFP) and yellow fluorescent protein (YFP) images. Cells were maintained on a microscope at 37 °C with a continuous supply of a mixture of 95% air and 5% carbon dioxide using a stage-top incubator (Tokai Hit, Shizuoka, Japan). Image analysis was performed using MetaMorph (Molecular Devices, Sunnyvale, CA, USA). The YFP/CFP emission ratio was calculated pixel-by-pixel using YFP and CFP images.

### Western blotting

Cells lysed with RIPA buffer were separated by sodium dodecyl sulfate polyacrylamide gel electrophoresis, and electrotransferred onto polyvinylidene fluoride membranes. After overnight incubation with primary antibody at 4 °C, the membranes were incubated with HRP-conjugated anti-mouse (GE Healthcare, Buckinghamshire, England) or anti-rabbit (GE Healthcare) secondary antibody for 1 h at room temperature, followed by addition of ECL prime (GE Healthcare) to detect bands using Image Quant LAS4000mini (GE Healthcare). Primary antibodies were detected against PFK-M (Abcam), PFK-L (Abcam), synaptopodin^14^, cleaved caspase-3 (Cell Signaling, Beverly, MA, USA), caspase-3 (Santa Cruz Biotechnology, Dallas, TX, USA) and actin (Sigma). All blots were run under the same experimental conditions. Each image was obtained at single time point and was not combined into a single image. Full length gels and blots for each figure are shown in Supplemental Figure 2.

### Scratch Anssay

FBS concentration was reduced to 0.5% 1 day before the assay. Confluent cells on six-well plates were scratched with a 200 μl sterile pipette, and loosely adherent cells and debris removed by washing with growth medium. Migrated podocytes or mesangial cells were counted when these cells crossed into scratched area. Images were obtained by BZ-X710 (Keyence, Osaka, Japan) at several time points. By comparing the images from 0 and 24 h, the distance of each scratch closure on the basis of the distances were measured^29^. Alternatively, relative ratio of cell migration in the presence of either antimycin or 2-DG/pyruvate to that in control condition was also determined to compare between podocytes and mesangial cells. The number of moved cells treated with either antimycin or 2-DG/pyruvate was divided by that with control condition. “Control” indicates a condition with normal condition medium.

### Immunofluorescence for cultured cells

Cells seeded on collagen-coated culture slides were fixed with 4% paraformaldehyde (PFA), permeabilized with 0.5% Triton X-100 in PBS for 5 min, and incubated for 1 h at room temperature with Alexa Fluor 488 Phalloidin (Invitrogen). After the slides were washed extensively with PBS, coverslips were mounted on slides with Vecta Shield (Vector, Burlingame, CA, USA). Mitochondria were visualized with MitoTracker (Life Technologies, Waltham, MA, USA) before fixation.

### Quantification of lamellipodia

Whole-cell perimeters and perimeters with adjacent lamellipodia (an actin-rich fringe with fluorescence intensity gradually declining with the distance from the edge) of fixed, phalloidin-stained cells were traced using MetaMorph line function. Lamellipodia length and area were quantified as described^30^.

### Apoptosis

Apoptotic cells were detected using an *In situ* Apoptosis Detection Kit (Takara, Shiga, Japan). Cells were fixed with 4% PFA and incubated in 70% ethanol at −80 °C overnight. After permeabilization with 0.1% Triton X-100, fragmented DNA was labeled by TdT enzyme for 120 min. The cells were mounted in Vecta Shield Mounting Medium with DAPI (Vector).

### Quantitative polymerase chain reaction (qPCR)

Cellular RNA was purified with RNeasy Mini Kit (Qiagen, Chatsworth, CA, USA), and first strand cDNA was synthesized using ReverTra Ace qPCR RT Kit (Toyobo, Osaka, Japan). Primers for synaptopodin were 5′-TTCCGAGTGGCATCCTTAAGTC-3′ (forward) and 5′-GCTGCTGCTTGGTAGGTTCA-3′ (reverse), and primers for 18S rRNA were 5′-AGGGGAGAGCGGGTAAGAGA-3′ (forward) and 5′-GGACAGGACTAGGCGGAACA-3′ (reverse). Amplification was performed in a Step One Plus thermal cycler (Applied Biosystems). The amplification program consisted of an initial denaturation at 95 °C for 10 min, followed by 40 cycles of denaturation at 95 °C for 15 sec, annealing at 58 °C for 10 sec, and extension at 72 °C for 1 min. Synaptopodin expression level in each sample was normalized relative to that of 18S rRNA.

### *In vivo* study

All animal experiments were performed in accordance with the Animal Experimentation Committee of Kyoto University and Tanabe R&D Service CO Ltd (Osaka, Japan). Mitochondria and foot process morphology in mouse podocytes were ultrastructurally analyzed by transmission electron microscopy (Hitachi S4700, Tokyo, Japan) at a magnification of ×4000. At least total 280 mitochondria and 160 foot processes were examined in three 20-week-old male C57Bl6 wild type mice and three 20-week-old male eNOS knockout mice (BKS.129P2(Cg)-Nos3/J) (Jackson Laboratories, Bar Harbor, ME, USA). The longest mitochondrial diameter and the shortest FP length were measured. For immunofluorescence for nephrin and PFK-L, the tissue samples were incubated with 10 mM citrate buffer (pH 6.0) at 50–90 °C for 30 min for retrievals and subsequently incubated overnight at 4 °C with primary antibodies, including goat anti-Nephrin (R&D Systems, Minneapolis, MN) and rabbit anti-PFKL. Then, sections were incubated with Alexa Fluor 488-labeled anti-rabbit IgG (Invitrogen) and Alexa Fluor 568-labeled anti-goat IgG (Invitrogen) for 1 h at room temperature, mounted with Vectashield anti-fade mounting medium (Vector Labs, Burlingame, CA) and finally examined by confocal microscopy (Leica TCS SP8; Leica Microsystems, Tokyo, Japan).

### Statistical analysis

All values are presented as mean ± SD. Statistical analysis was performed with ANOVA using Tukey’s method to compare groups or two-tailed t test. A level of p < 0.05 was considered statistically significant.

## Additional Information

**How to cite this article**: Ozawa, S. *et al.* Glycolysis, but not Mitochondria, responsible for intracellular ATP distribution in cortical area of podocytes. *Sci. Rep.* **5**, 18575; doi: 10.1038/srep18575 (2015).

## Supplementary Material

Supplemental Figure

## References

[b1] Verma R. *et al.* Nephrin ectodomain engagement results in Src kinase activation, nephrin phosphorylation, Nck recruitment, and actin polymerization. J Clin Invest. 116, 1346–1359 (2006). 16543952 10.1172/JCI27414 PMC1401486

[b2] Shiraishi T. *et al.* Glycolysis is the primary bioenergetic pathway for cell motility and cytoskeletal remodeling in human prostate and breast cancer cells. Oncotarget. 6, 130–143 (2015). 25426557 10.18632/oncotarget.2766 PMC4381583

[b3] Buchakjian M. R. & Kornbluth S. The engine driving the ship: metabolic steering of cell proliferation and death. Nat Rev Mol Cell Biol. 11, 715–727 (2010). 20861880 10.1038/nrm2972

[b4] Vander Heiden M. G., Cantley L. C. & Thompson C. B. Understanding the Warburg effect: the metabolic requirements of cell proliferation. Science. 324, 1029–1033 (2009). 19460998 10.1126/science.1160809 PMC2849637

[b5] Bolanos J. P., Peuchen S., Heales S. J., Land J. M. & Clark J. B. Nitric oxide-mediated inhibition of the mitochondrial respiratory chain in cultured astrocytes. J Neurochem. 63, 910–916 (1994). 7519665 10.1046/j.1471-4159.1994.63030910.x

[b6] Pellerin L. & Magistretti P. J. Glutamate uptake into astrocytes stimulates aerobic glycolysis: a mechanism coupling neuronal activity to glucose utilization. Proc Natl Acad Sci USA 91, 10625–10629 (1994). 7938003 10.1073/pnas.91.22.10625 PMC45074

[b7] De Bock K. *et al.* Role of PFKFB3-driven glycolysis in vessel sprouting. Cell. 154, 651–663 (2013). 23911327 10.1016/j.cell.2013.06.037

[b8] Geudens I. & Gerhardt H. Coordinating cell behaviour during blood vessel formation. Development. 138, 4569–4583 (2011). 21965610 10.1242/dev.062323

[b9] Potente M., Gerhardt H. & Carmeliet P. Basic and therapeutic aspects of angiogenesis. Cell. 146, 873–887 (2011). 21925313 10.1016/j.cell.2011.08.039

[b10] Abe Y., *et al.* Bioenergetic characterization of mouse podocytes. Am J Physiol Cell Physiol. 299, C464–476 (2010). 20445170 10.1152/ajpcell.00563.2009 PMC2928644

[b11] Imamura H. *et al.* Visualization of ATP levels inside single living cells with fluorescence resonance energy transfer-based genetically encoded indicators. Proc Natl Acad Sci USA 106, 15651–15656 (2009). 19720993 10.1073/pnas.0904764106 PMC2735558

[b12] Almeida A., Almeida J., Bolanos J. P. & Moncada S. Different responses of astrocytes and neurons to nitric oxide: the role of glycolytically generated ATP in astrocyte protection. Proc Natl Acad Sci USA 98, 15294–15299 (2001). 11742096 10.1073/pnas.261560998 PMC65023

[b13] Mundel P., Gilbert P. & Kriz W. Podocytes in glomerulus of rat kidney express a characteristic 44 KD protein. J Histochem Cytochem. 39, 1047–1056 (1991). 1856454 10.1177/39.8.1856454

[b14] Mundel P. *et al.* Synaptopodin: an actin-associated protein in telencephalic dendrites and renal podocytes. J Cell Biol. 139, 193–204 (1997). 9314539 10.1083/jcb.139.1.193 PMC2139823

[b15] Sheng Z. H. & Cai Q. Mitochondrial transport in neurons: impact on synaptic homeostasis and neurodegeneration. Nat Rev Neurosci. 13, 77–93 (2012). 22218207 10.1038/nrn3156 PMC4962561

[b16] King M. J., Atwood H. L. & Govind C. K. Structural features of crayfish phasic and tonic neuromuscular terminals. J Comp Neurol. 372, 618–626 (1996). 8876457 10.1002/(SICI)1096-9861(19960902)372:4<618::AID-CNE9>3.0.CO;2-7

[b17] Nguyen P. V., Marin L. & Atwood H. L. Synaptic physiology and mitochondrial function in crayfish tonic and phasic motor neurons. J Neurophysiol. 78, 281–294 (1997). 9242280 10.1152/jn.1997.78.1.281

[b18] Verstreken P. *et al.* Synaptic mitochondria are critical for mobilization of reserve pool vesicles at Drosophila neuromuscular junctions. Neuron. 47, 365–378 (2005). 16055061 10.1016/j.neuron.2005.06.018

[b19] Lee C. W. & Peng H. B. The function of mitochondria in presynaptic development at the neuromuscular junction. Mol Biol Cell. 19, 150–158 (2008). 17942598 10.1091/mbc.E07-05-0515 PMC2174173

[b20] Simsek T. *et al.* The distinct metabolic profile of hematopoietic stem cells reflects their location in a hypoxic niche. Cell Stem Cell. 7, 380–390 (2010). 20804973 10.1016/j.stem.2010.07.011 PMC4159713

[b21] Suda T., Takubo K. & Semenza G. L. Metabolic regulation of hematopoietic stem cells in the hypoxic niche. Cell Stem Cell. 9, 298–310 (2011). 21982230 10.1016/j.stem.2011.09.010

[b22] Yu W. M. *et al.* Metabolic regulation by the mitochondrial phosphatase PTPMT1 is required for hematopoietic stem cell differentiation. Cell Stem Cell. 12, 62–74 (2013). 23290137 10.1016/j.stem.2012.11.022 PMC3632072

[b23] Birket M. J. *et al.* A reduction in ATP demand and mitochondrial activity with neural differentiation of human embryonic stem cells. J Cell Sci. 124, 348–358 (2011). 21242311 10.1242/jcs.072272 PMC3021997

[b24] Asanuma K. *et al.* Synaptopodin regulates the actin-bundling activity of alpha-actinin in an isoform-specific manner. J Clin Invest. 115, 1188–1198 (2005). 15841212 10.1172/JCI23371 PMC1070637

[b25] Aronchik I. *et al.* Actin reorganization is abnormal and cellular ATP is decreased in Hailey-Hailey keratinocytes. J Invest Dermatol. 121, 681–687 (2003). 14632182 10.1046/j.1523-1747.2003.12472.x

[b26] Nakayama T. *et al.* Endothelial von Willebrand factor release due to eNOS deficiency predisposes to thrombotic microangiopathy in mouse aging kidney. Am J Pathol. 176, 2198–2208 (2010). 20363914 10.2353/ajpath.2010.090316 PMC2861085

[b27] Ueda S. *et al.* ENOS deficiency causes podocyte injury with mitochondrial abnormality. Free Radic Biol Med. 87, 181–192 (2015). 26119782 10.1016/j.freeradbiomed.2015.06.028

[b28] Shankland S. J., Pippin J. W., Reiser J. & Mundel P. Podocytes in culture: past, present, and future. Kidney Int. 72, 26–36 (2007). 17457377 10.1038/sj.ki.5002291

[b29] Liang C. C., Park A. Y. & Guan J. L. *In vitro* scratch assay: a convenient and inexpensive method for analysis of cell migration *in vitro*. Nat Protoc. 2, 329–333 (2007). 17406593 10.1038/nprot.2007.30

[b30] Yang C. *et al.* Novel roles of formin mDia2 in lamellipodia and filopodia formation in motile cells. PLoS Biol. 5, e317 (2007). 18044991 10.1371/journal.pbio.0050317 PMC2229861

