# Supplementary material for: Glycolysis, but not Mitochondria, responsible for intracellular ATP distribution in cortical area of podocytes
Source: Sci Rep. 2015 Dec 18;5:18575. doi: 10.1038/srep18575 (PMC4683464; doi:10.1038/srep18575)
Supplement: Supplemental Figure [file srep18575-s1.pdf]

Glycolysis, but not Mitochondria, responsible for intracellular ATP distribution in cortical area of podocytes

Shota Ozawa[1,2], Shuko Ueda[3], Hiromi Imamura[4], Kiyoshi Mori[1], Katsuhiko Asanuma[1], Motoko Yanagita[1,5] & Takahiko Nakagawa[1,*]

[1]TMK project, Medical Innovation Center, Kyoto University, Kyoto, Japan

[2]Pharmacology Research Laboratories II, Mitsubishi Tanabe Pharma Corporation, Saitama, Japan

[3]Department of Internal Medicine, Teikyo University School of Medicine, Tokyo, Japan

[4]Laboratory of Functional Biology, Graduate School of Biostudies, Kyoto University, Kyoto, Japan

[5]Department of Nephrology, Kyoto University Graduate School of Medicine, Kyoto, Japan

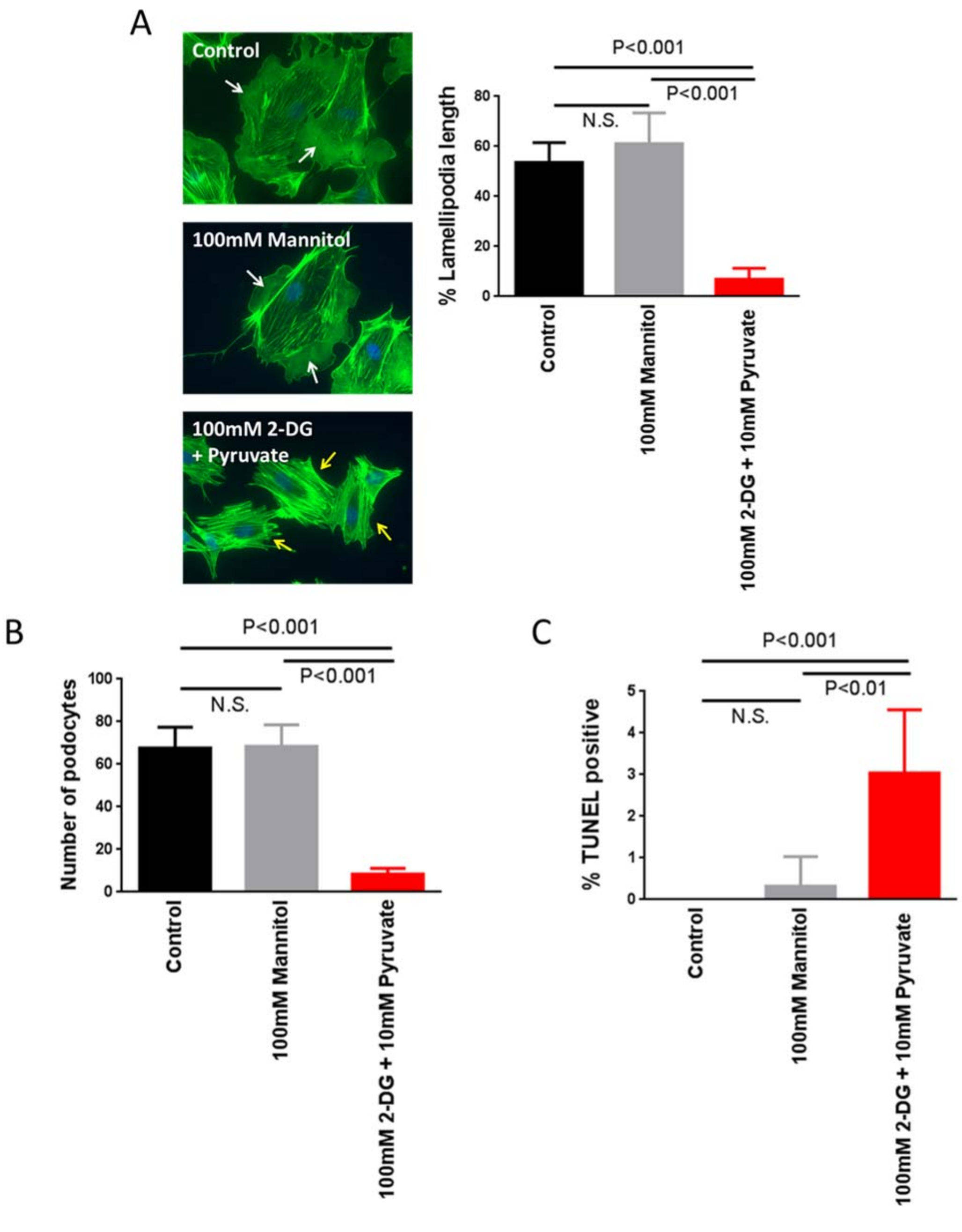

Supplemental Figure 1: Hyperosmolarity has no effects on lamellipodia formation, migratory ability and cell survival in the cultured podocytes. At 24 h, the lamellipodia formation (white arrow) is maintained with either control or 100mM Mannitol while it is not seen with 100mM 2-DG with 10mM Pyruvate (yellow arrow) (A). Likewise, 100mM Mannitol has no effects on podocyte migration in scratch assay (B) and the number of TUNEL-positive cells (C) in contrast to 100mM 2-DG with 10mM Pyruvate.

Fig. 1C

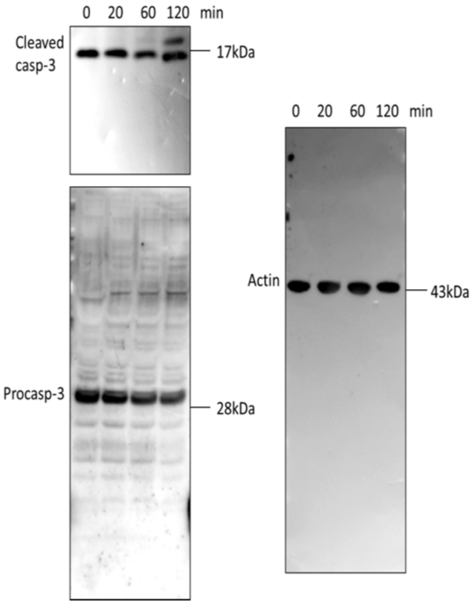

Fig. 3H

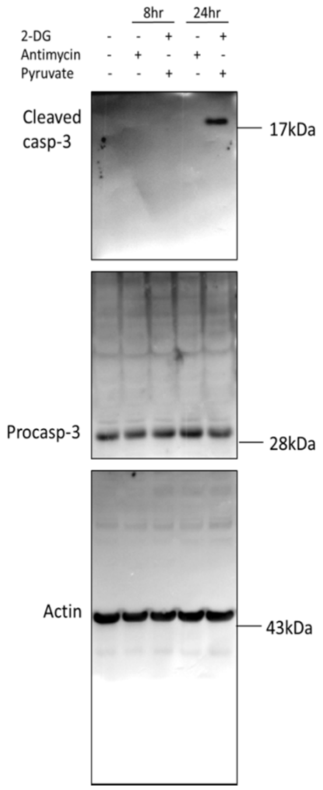

Fig. 4A

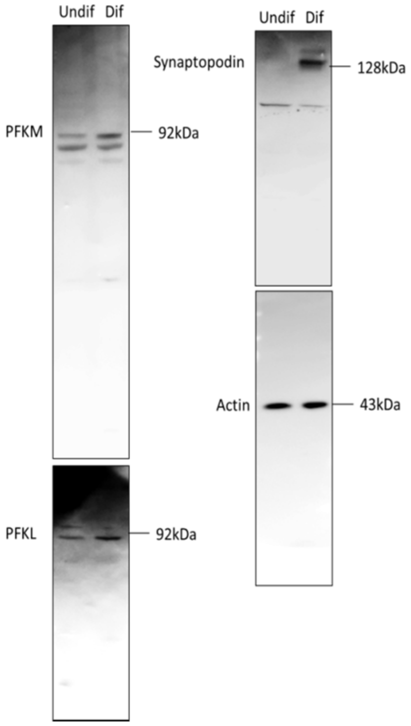

Fig. 4B

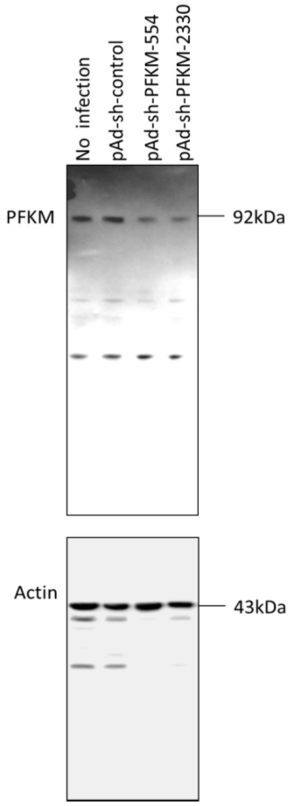

Fig. 4C

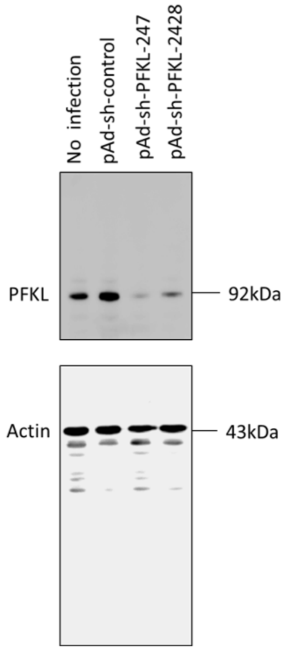

Supplemental Figure 2: Original blots for each figure are shown.
